# The impact of using AI-powered voice-to-text technology for clinical documentation on quality of care in primary care and outpatient settings: a systematic review

**DOI:** 10.1016/j.ebiom.2025.105861

**Published:** 2025-07-21

**Authors:** Ahmed Alboksmaty, Reham Aldakhil, Benedict W.J. Hayhoe, Hutan Ashrafian, Ara Darzi, Ana-Luisa Neves

**Affiliations:** a Institute of Global Health Innovation (IGHI), Department of Surgery and Cancer, Imperial College London, St Mary’s Campus, Norfolk Place, London W2 1PG, UK; b Department of Primary Care and Public Health, School of Public Health, Imperial College London, 90 Wood Ln, London W12 0BZ, UK

**Keywords:** Primary care, Outpatient, Artificial intelligence, AI scribes, Digital health, Quality of care

## Abstract

**Background:**

AI-powered Voice-to-text Technology (AIVT) offers a promising solution to reduce clinicians’ documentation burden during consultations, allowing more focus on patient interaction. This systematic review assesses AIVT’s impact on care quality in primary care and outpatient settings, focusing on seven components: effectiveness, efficiency, safety, patient-centredness, timeliness, equity, and integration.

**Methods:**

A systematic search of five databases (Medline, Embase, Global Health, CINHAL, Scopus) was conducted for studies published up to September 20, 2024. Studies were included if they assessed the use of AIVT for medical documentation in primary care or outpatient settings, compared to manual or non-AI documentation methods, and reported outcomes relevant to the seven quality components. A narrative synthesis was conducted; meta-analysis was unfeasible due to study heterogeneity.

**Findings:**

Of 1924 papers, nine studies were included (n = 524 healthcare professionals, n = 616 patients, 1069 consultations). Most (n = 7) were from the USA, with others in Bangladesh and the Philippines. All studies assessing effectiveness, patient-centredness, and efficiency (n = 9, 6, and 5, respectively) reported improvements, including faster documentation, reduced administrative burden, and enhanced patient-provider interaction. Safety findings were inconclusive; three of six studies raised concerns. Four studies highlighted seamless AIVT integration with Electronic Health Records, improving service timeliness. Three studies identified equity issues, referring to limited diversity and controlled simulation settings.

**Interpretation:**

AIVT tools enhance documentation efficiency and patient-centred care, but concerns over transcription errors and generalisability warrant further testing in large-scale, diverse real-world settings.

**Funding:**

This study was supported by the 10.13039/501100000272National Institute for Health and Care Research (NIHR) North-West London Patient Safety Research Collaboration (NIHR NWL PSRC, Ref. NIHR204292), with infrastructure support from the 10.13039/501100013342NIHR Imperial Biomedical Research Centre.

**Research in context**

**Evidence before this study**

Healthcare providers, including General Practitioners (GPs) are increasingly burdened with multitasking during patient consultations, with accurate documentation being a critical yet attention-taking responsibility. While digital health tools have been introduced to enhance care quality, the need to focus on computer-based manual documentation often disrupts direct patient-provider interactions. The advent of Artificial Intelligence (AI) tools presents a potential solution, particularly through AI-powered voice-to-text (AIVT) tools, which automate documentation and alleviate administrative strain. These tools have been widely implemented in hospital settings for dictating clinical notes, discharge summaries, and ward-round documentation. Their use in individual consultations, whether in primary care or outpatient settings, is also expanding. However, evidence of AIVT’s impact on care quality remains limited. Specifically, little is known about how these tools influence key quality domains, including effectiveness, efficiency, timeliness, equity, safety, patient-centredness, and care integration. This systematic review seeks to address this gap by summarising the evidence of AIVT’s influence on the quality of primary care and outpatient consultations, providing a comprehensive assessment of its implications for care delivery.

**Added value of this study**

The findings of this review indicate that AIVT tools can enhance care quality in primary care and outpatient consultations by improving effectiveness, efficiency, and patient-centredness. Existing literature suggests that AIVT-generated documentation is comparable to, and in some cases surpasses, manual documentation quality while reducing administrative burden and enabling greater focus on patient interactions. However, regarding safety, studies have reported concerns about transcription inaccuracies, which may pose patient safety risks and necessitate provider review before finalising medical notes in patient records. Equity also remains insufficiently assessed, as most studies involved highly educated, native English-speaking participants or were conducted in controlled settings, limiting generalisability to diverse patient populations in real-world practice. The literature also suggests that integrating AIVT tools within existing health systems is feasible, though further research is needed to evaluate their implementation across varied healthcare settings.

**Implications of all the available evidence**

Our findings highlight the need for standardised policies and regulations to ensure the safe and effective integration of AIVT into clinical practice, addressing ethical concerns such as data security, patient consent, and accountability. While AIVT can enhance efficiency, potential transcription inaccuracies necessitate provider oversight to uphold patient safety. Research should prioritise rigorous real-world testing across diverse populations, moving beyond technical development to assess its impact on patient outcomes, provider experiences, and healthcare workflows. Transparent reporting is essential to mitigate publication bias and ensure a balanced evaluation of AIVT’s role in primary care. Additionally, successful implementation requires adequate clinician training, clear workflow adaptations, and strategies to manage potential risks, to ensure seamless adoption into healthcare settings.

## Introduction

The integration of Artificial Intelligence (AI) into healthcare has introduced transformative tools designed to address contemporary challenges, such as increasing care complexity, service fragmentation, and workforce shortages, that potentially reshape clinical workflows and offer new models of care.^1^ Among these advancements, one particularly promising application is AI-powered Voice-to-text Technology (AIVT), which aims to alleviate the burden of documentation faced by healthcare professionals during medical consultations.^2^ This technology offers a streamlined solution for a task that often requires clinicians to multitask - balancing patient interaction, Electronic Health Records (EHRs) navigation, and accurate notetaking.^3^

While computers and electronic technologies improve access to patients’ medical histories and support decision-making,^4^ they are often seen as competing for attention during consultations, particularly when navigating EHR and typing documentation.^5^ This dynamic has led both patients and providers to seek ways to minimise the attention required for computers, allowing greater focus on active patient communication.^6^ By automating documentation, AIVT has the potential to reduce administrative burdens, enabling technology to step back from creating a sense of isolation and instead act as an advocate for patient-centred communication, ultimately fostering more meaningful interactions in practice.^7^^,^^8^ Additionally, as EHRs become increasingly accessible to patients, AIVT-generated documentation may enhance trust by providing more accurate and comprehensible patient-facing notes.^9^

There is no standardised universal definition for AI. Since the term was first introduced in 1956, the scientific community has broadly described AI as the simulation of human intelligence by computational systems, the creation of intelligent machines, or the development of self-learning computers.^10^ For the purpose of this study, we have adopted the Royal College of General Practice’s (RCGP) definition, describing AI as “*technologies with the ability to perform tasks that would otherwise require human intelligence*”.^11^ These technologies may involve, but are not limited to, machine learning, Natural Language Processing (NLP), speech analysis, and other innovative methods.^11^ Powering voice-to-text tools with these technologies (i.e., AIVT) offers an opportunity to redefine the documentation process in a typical primary care or outpatient clinic consultation.^12^^,^^13^

There is no commonly agreed-upon structure for defining AIVT in the literature, while it is proposed to improve patient outcomes, address clinician burnout, and enhance consultation efficiency.^14^^,^^15^ It is crucial to assess how using AIVT may influence the quality of care, identified by its six dimensions as defined by the Institute of Medicine (IOM) (i.e., safety, effectiveness, efficiency, patient-centredness, timeliness, and equity),^16^ in addition to the seventh commonly stated dimension by the World Health Organization WHO, which is the integration of care.^17^

The impact of AIVT may vary depending on the context of its application, whether scheduled appointments in clinics, emergency care, operation theatres, or inpatient hospital wards. Additionally, the mode of implementation, whether used for documenting one-person dictations, individual patient-provider interactions, or group-team discussions, can influence its effectiveness.^18^, ^19^, ^20^

Previous reviews have primarily adopted a technology-centred perspective, focusing on the development, technical features, and enabling or limiting factors of AIVT,^20^, ^21^, ^22^ or more broadly, AI technologies in healthcare, rather than appraising their implementation in practice.^23^^,^^24^ This systematic review aims to assess the evidence on using AIVT to document individual patient-provider medical consultations, specifically in primary care and outpatient clinic settings, and its impact on care quality. The review seeks to provide a comprehensive understanding of the potential of this technology to enhance healthcare service delivery and improve patient outcomes.

## Methods

This systematic review was designed and reported guided by the Preferred Reporting Items for Systematic Reviews and Meta-Analyses (PRISMA) 2020 Checklist.^25^ We registered the study protocol with the International Prospective Register of Systematic Reviews (CRD42024594657).

### Search strategy

A comprehensive search was conducted across five databases: Medline, Embase, Global Health, CINHAL, and Scopus. Engineering and technology-focused databases, such as the Association for Computing Machinery (ACM) Digital Library and IEEE Xplore, were not included in the search, as their primary focus often leans toward technical AI model development. The chosen databases, however, provide extensive coverage of relevant literature, including studies overlapping with those typically found in such technical databases, ensuring that our review captured the necessary scope of evidence.

Each database was independently searched on September 20, 2024, and included all published studies up to that date, following agreement on subject headings and keywords to cover two main concepts: voice recognition software and artificial intelligence. To ensure a comprehensive search strategy, we consulted a medical librarian during its development. We also reviewed search terms from previous relevant reviews and considered them in our strategy. A full list of search terms is provided in Appendix 1. Reference lists of previous relevant reviews were also screened. No restrictions on publication date or language were applied to the search.

### Inclusion and exclusion criteria

We included studies that assessed the use of AIVT in primary care or outpatient clinic settings, specifically for documenting two-way medical consultations between a healthcare professional and a patient. Studies centred on single-person dictation or group discussions, such as those in hospital wards or operating theatres, were not considered. Both quantitative and qualitative studies were eligible if they reported outcomes relevant to the quality domains. A detailed list of the inclusion and exclusion criteria, based on the PICO-S framework (Population, Intervention, Comparator, Outcomes, and Study Type),^26^ is provided in Table 1.

**Table 1:** Inclusion and exclusion criteria.

Items/Criteria	Inclusion	Exclusion
Population (and setting)	• Medical consultations in primary care or outpatient hospital clinics, primarily involving individual patient consultations, whether virtual or in-person consultations. • Consultations conducted by any healthcare professional (e.g., doctors, nurses, social prescribers, clinical pharmacists, physiotherapists). • Studies conducted in healthcare systems in any country.	• Medical reviews in hospital wards, hearing clinics, or speech therapy settings. • Studies that exclusively focused on non-healthcare settings or unrelated industries. • Emergency calls and triaging in emergency departments will be excluded as it represents unique circumstances. • Studies that assess the technology in operation theatres will be excluded, as in different settings.
Intervention	• Use of AI-powered voice-to-text technology for medical documentation or scribing in face-to-face, telephone, or video consultations. Pre-recorded, re-enacted, and live consultations will be included. • Studies involving technologies that are at least at the “proof of concept” stage or beyond (e.g., in clinical or pilot use).	• Exclusive use of traditional, non-AI-powered voice-to-text software for medical scribing. • Use of AI technologies (e.g., NLP) to analyse manually written consultation scripts or scripts not generated by AI-powered tools. • Exclusive use of one-person dictation for documentation, whether during or after a medical consultation.
Comparator	• Consultations documented manually or using non-AI-powered technologies. • Studies comparing different AI-powered voice-to-text technologies.	• Studies comparing AI technologies used to analyse medical scripts without involving voice-to-text technology
Outcomes	• Studies reporting outcomes aligned with **the six quality domains** (patient-centeredness, clinical effectiveness, efficiency, timeliness, equity, and safety). Examples of relevant outcomes include transcription accuracy (safety), documentation speed (effectiveness), user satisfaction (patient-centeredness), ease of integration into clinical workflows (efficiency), support for clinical decision-making (timeliness), and perceptions of equity in the delivery of care. This is in addition to the seventh domain highlighted by the WHO, integration of services. • Studies assessing the impact of AI-powered voice-to-text technology on **patient outcomes** (such as patient safety, and continuity of care) or the **work dynamics** (such as consultation time, and the ease of drafting referrals)	• Studies focused on AI technologies for hearing aids or speech therapy. • Studies reporting general outcomes without specific analysis of AI-powered voice-to-text technologies, even if such technologies were used in the study. • Studies focusing on theoretical models, simulations, or AI technologies that have not been tested in actual consultations or clinical settings.
Study type	• Randomised controlled trials, cluster-randomised trials, quasi-experimental studies, case-control studies, cohort studies, cross-sectional studies, relevant case reports, and cost-effectiveness studies. • Studies used qualitative, quantitative, or mixed methods approach.	• Incomplete studies, commentary articles, systematic reviews, interim reports, scoping reviews, case series, opinion pieces, conference abstracts, or trial protocols.

### Data screening

Duplicates were identified and removed initially through EndNote^27^ and then further checked using Covidence,^28^ where the data screening was processed. Two researchers with relevant experience (AA and RA) independently screened the studies in two phases: first by reviewing titles and abstracts, followed by a full-text review for the shortlisted studies. Any disagreements were initially resolved through discussion between the two researchers. If a disagreement persisted, a third senior researcher (ALN) was consulted to reach a consensus. Inter-rater agreement for each screening phase was assessed using Cohen’s κ, with a score above 0.6 considered substantial, based on previous literature, to proceed to the next phase.^29^

### Data extraction

The research team agreed upon a set of data extraction items (Appendix 2) designed to capture the technical and descriptive aspects of the included studies. Two researchers (AA and RA) independently extracted data from the included studies and mapped the findings to the relevant quality domains. They subsequently reviewed the extracted data together and discussed the information identified for each domain. A senior researcher (ALN) conducted a final review of the data extraction table and was available to provide guidance in case of any discrepancies, although none were identified.

The data extraction process prioritised information on the adoption and the use of the technology in practice rather than the technical aspects of AI model development. Relevant details concerning the quality domains were further discussed among the team, even when not explicitly linked to these domains by the original authors of the included studies.

### Quality assessment

Given the diverse methodologies of the studies included in this review, we utilised the Mixed Methods Appraisal Tool (MMAT) to comprehensively and fairly evaluate the methodological quality of each study based on its design.^30^ The MMAT provides tailored evaluation criteria for various study methodologies, including quantitative, qualitative, and mixed methods. Instead of using a scoring system, the MMAT focuses on assessing the methodological rigour and implementation processes of each study, making it particularly suitable for the complex implementation methodologies represented in this review.

Using MMAT is considered a deviation from the original protocol, which proposed using the Newcastle-Ottawa Scale (NOS) and Critical Appraisal Skills Programme (CASP) tools.^31^^,^^32^ This change was made after discussions among the co-authors due to the methodological heterogeneity of the included studies, which made the MMAT a more comprehensive tool to ensure consistency in assessment across different study designs. In this review, we use the MMAT specifically to assess methodological quality rather than to provide a direct measure of risk of bias, recognising that these are related but distinct concepts.

### Data analysis

Due to the heterogeneity of study designs and outcomes, a meta-analysis was not conducted. We, therefore, adopted a narrative synthesis method, guided by a thematic analysis that used the seven quality domains as predefined analytical themes,^33^ to explore the impact of using AIVT for medical documentation on the quality of care. To enable a coherent synthesis, we used a convergent integrated approach in which quantitative findings were described in narrative form, focusing on their implications and contextual relevance to the quality domains.^34^ This allowed us to integrate evidence from both quantitative and qualitative studies in a coherent and meaningful manner. Two researchers (AA & RA) reviewed and discussed the extracted data to identify information relevant to each of the seven quality domains. Table 2 presents the original definitions of the seven quality domains, as adopted from the IOM and WHO,^16^^,^^17^ alongside contextualised definitions tailored to the purpose of this study. The findings were then thoroughly discussed with the other researchers to reach a consensus on the main outcomes.

**Table 2:** Contextualised definitions of the quality domains.

Quality domain	IOM definitions^16^ + Integration^17^	Contextualised definitions
Effectiveness	Providing care based on evidence to those who benefit, avoiding ineffective care	Assessing whether digital scribes are developed and fit for their intended purpose, improving documentation quality and enabling more personalised care that help clinical decision-making, and are used appropriately across relevant settings.
Efficiency	Avoiding waste of resources, effort, and time.	Determining whether digital scribes reduce clinician workload and administrative burden without adding new technical, clinical, or practical difficulties.
Patient-centredness	Respecting and responding to patient preferences, needs, and values	Considering whether digital scribes support or disrupt clinician–patient communication, trust, and patient comfort and general experience during medical consultations.
Timeliness	Reducing waits and delays in care	Evaluating whether digital scribes efficiently reduce documentation time and support more efficient consultations, follow-up processes, or care coordination.
Safety	Avoiding harm to patients from care intended to help	Ensuring that AIVT-generated documentation does not introduce clinical or data-related risks, such as transcription errors or privacy breaches that may risk patient safety.
Equity	Ensuring care quality does not vary due to personal characteristics	Exploring whether digital scribes perform consistently across diverse patient populations and do not exacerbate disparities in care delivery or documentation.
Integration	Coordinated delivery of health services across different levels and settings of care, including integration of systems and tools to support continuity.	Evaluating whether digital scribes integrate seamlessly into existing clinical systems (e.g., EHRs) and workflows to enhance coordinated care, data sharing, and continuity across settings.

### Statistics

We did not conduct any statistical analysis as part of our methodology.

### Ethics

This study is a systematic review of published data on the use of AIVT tools in primary care and outpatient settings. As such, ethical approval was deemed unnecessary, in accordance with advice from the Research Governance and Integrity Team at Imperial College London.

### Role of funders

The views expressed in this publication are those of the authors and not necessarily those of the NIHR or the Department of Health and Social Care. The funders had no role in study design, data collection and analysis, decision to publish, or writing the manuscript.

## Results

**Fig. 1:**
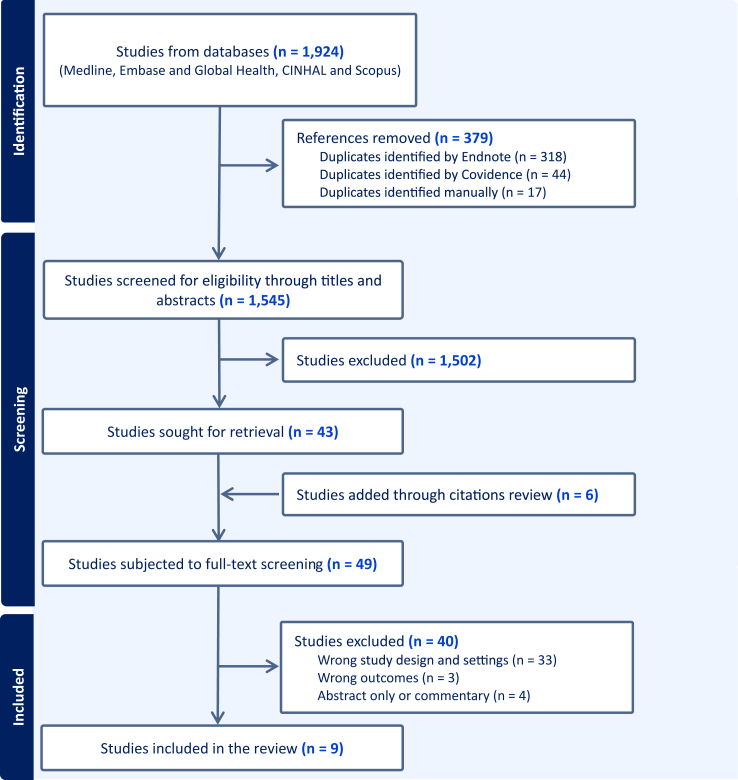
PRISMA flow chart clarifying the screening process and outcomes.

The initial search across five databases identified 1924 results. After removing 379 duplicates, 1502 records were excluded through title and abstract screening. Forty-nine studies were reviewed in full text, including 43 shortlisted from the initial screening and six additional studies found through citation review. Nine studies met the inclusion criteria. Inter-rater agreement was calculated with Cohen’s κ scores of 0.75 for title/abstract screening and 0.62 for full-text review.^29^ Fig. 1 shows the detailed screening process.

### Summary of included studies

Most included studies (n = 7) were conducted in the USA,^7^^,^^35^, ^36^, ^37^, ^38^, ^39^, ^40^ with one in Bangladesh^41^ and another in the Philippines.^42^ All were published between 2018 and 2024, reflecting recent advancements in AIVT technologies. A quantitative approach was used in all studies, with two studies employing the System Usability Score (SUS) to assess the intervention.^41^^,^^42^

The studies collectively involved 524 healthcare professionals and 616 patients, evaluating 1069 medical consultations. Most healthcare professionals were General Practitioners (GPs) or advanced primary care providers. Two studies had patient category restrictions: one focused on diabetics,^41^ and the other excluded psychiatry and paediatrics consultations, being hard to simulate for research purposes.^40^

Five studies were conducted in, or simulated, primary care,^7^^,^^37^, ^38^, ^39^^,^^42^ two in outpatient clinics,^35^^,^^41^ and two included both settings.^36^^,^^40^ Four studies assessed interventions in simulated environments.^37^^,^^39^^,^^40^^,^^42^ No study specifically evaluated AIVT technology in telephone or virtual medical encounters. The characteristics and overview of the included studies are summarised in Table 3.

**Table 3:** Summary of the included studies.

Study (Year)	Study period	Country	Study design	Setting	Participants	Comparator to AIVT use	Outcome measures	Key findings
Goss et al. (2019)^35^	Sep 2016 - Mar 2017	USA	Cross sectional survey	Hospital settings, including OP clinics	245 (200 doctors; 31 PA; 12 Nurses; 1 social worker; 1 neuropsychologist)	Not explicitly stated; comparisons made among clinician-reported outcomes based on AIVT use	• Clinician satisfaction • Perceived accuracy • Editing time • Efficiency	• Clinician satisfaction: 78.8% (associated with perceived efficiency) • 75.5% of users estimated <10 errors per dictation, and 19.6% estimated half or more of the errors were clinically significant. • Increased efficiency (77.2%) by AIVT use; however, 21.2% of clinicians reported spending 25% or more of their documentation time on editing. • Error Impact on Satisfaction: Clinicians who reported more >20 errors per document were 14 times more likely to be dissatisfied with AIVT use compared to those reporting <5 errors (p < 0.001).
Haberle et al. (2024)^36^	March to September 2022	USA	Prospective peer-matched controlled cohort study	Primary care and OP clinics	198 (99 intervention group, including 56 GPs, and 99 control group)	A peer-matched control group without AIVT	• Provider Engagement • Productivity • Efficiency • Patient Experience • Documentation Quality	• Positive trends in provider engagement among AIVT users • Statistically significant increase in productivity among AIVT users. (The average time spent on documentation per patient for AIVT users decreased from 5.3 min to 4.54 min (p < 0.001). The control group showed a non-significant decrease from 5.5 to 5.35 min) • AIVT users had a 4.69% increase in after-hours EHR activity (p < 0.05). The control group had a non-significant reduction in after-hours EHR activity by 0.945%. • AIVT users had a significant reduction in documentation deficiency rate (completion of notes within 24-h timeframe), from 8.6% to 6.3% (p < 0.001). The control group showed a slightly lower improvement rate (from 7.7% to 5.9%). • An expert internal audit team didn’t find any meaningful evidence of over-coding, missed risk-adjustment opportunity, or insufficient supporting documentation.
Islam et al. (2024)^41^	Not specified - End of 2023	Bangladesh	Usability evaluation study (using SUS)	OP clinics	23 (17 patients with diabetes and 6 doctors), conducted 102 consultations	Handwritten scribe and prescription	• Usability, accuracy of generated scribes and prescriptions	• The SUS score for the AIVT-generated scribes averaged 4.33/5, while it was 4.83/5 for prescriptions.
Kodish-Wachs et al. (2018)^37^	Not specified - Early 2018	USA	Retrospective comparative observational study	Primary Care	9 (2 doctors and 7 patients); recorded 34 simulated consultations	Comparing eight different AIVT tools with human transcription and note-taking as the gold standard	• Word Error Rate (WER) • Precision and accuracy • Recall • F1 (accuracy measure) score for clinical concepts	• WER among the eight AIVT tools ranged from 35% (best; MAVIS.V2) to 65% (worst; DeepSpeech) • Clinical concept recall rates ranged from 22% to 73% (MAVIS.V2 was the best in recall reflecting better information extraction) • The average F1 score was approximately 60% across all engines, with the best tool achieving around 73% recall and similar F1 performance. • Insertion and deletion errors contributed notably to the overall high WERs (some scenarios reached WER of 86%, with notable errors in capturing medication names)
Owens et al. (2) (2024)^38^	Apr-23	USA	Retrospective comparative observational study, and a cross-sectional survey	Primary care	83 (mainly GPs and primary care providers), and 240 monthly reports.	Traditional documentation methods during consultation (handwriting, dictation after consultations or typing)	• Provider burnout • Time efficiency • Documentation burden (defined as time outside of schedules hours per day.)	• Increase in AIVT use was associated with significantly less burnout. • Average documentation time per note in the pre-implementation period was 5.9 (±2.6) min, while after AIVT use was significantly reduced by 28.8% (1.8 min (95%CI: 1.4–2.2)), and time documenting outside of scheduled hours was significantly reduced by 11.8% (4 min (95%CI: 1–7.2)). • Percent contribution to the note by provider decreased by 33% (95% CI 24.2%–41.8%), while documentation length increased by an average of 542 characters (95%CI: 283.9–800.1).
Owens et al. (2024)^7^	Apr-23	USA	Prospective comparative observational study	Primary care	592 patients (primary care consultations)	Traditional documentation methods during consultation (handwriting, dictation after consultations or typing)	• Patient satisfaction	• Phase 1 (patients were aware of using an AIVT tool): they reported that the provider was more focused on them (75.4% (95%CI:70.4–80.3%; p < 0.001)), spent less time typing (78.8% (74.1–83.5%; p < 0.001)); felt more personable conversation (80.9% (76.4–85.4%; p < 0.001)). • Phase 2 (patients were blind of whether using an AIVT tool or not): there was no significance in their satisfaction between consultations where actually an AIVT was used and without.
Tran et al. (2023)^39^	Nov-21	USA	Retrospective comparative observational study	Primary care	5 GPs conducted 36 consultations, which were re-enacted by 2 graduate students for the purpose of the study.	Evaluation of two AIVT systems (Google and Amazon) on capturing clinically-relevant Non-Lexical Conversational Sounds (NLCS)	• WER for both tools • Accuracy in interpreting clinically relevant NLCS	• Overall accuracy: Google AIVT (11.8% WER) performed slightly better than Amazon’s (12.8% WER). • NLCS recognition: Both tools had high error rates with NLCS, with Google at 40.8% and Amazon at 57.2%. • Clinically relevant NLCS misinterpretation: extremely high misrecognition rates (94.7% for Google and 98.7% for Amazon). • Error Types: (1) Substitution: NLCS replaced with unrelated words (2) Deletion: Important NLCS omitted, affecting general understanding (3) Insertion: Unintended NLCS introduced, causing confusion
Wang et al. (2021)^40^	Not specified - mid 2020	USA	Prospective comparative observational study	Primary care and OP clinics	64 simulated primary care consultations (excluding psychiatry and paediatrics), recorded by 2 medical students	Traditional documentation methods (typing and dictation).	• Documentation speed (measured in words per minute) • Completion time of notes • Correlation between user experience over time and documentation speed.	• Documentation speed: AIVT tools achieved 2.7 times faster documentation for history sections compared to typing and dictation, and 2.17 times faster for physical examination sections compared to typing and 3.12 times faster than dictation. • A significant positive correlation between the number of encounters completed by an AIVT tool and the completion speed for history sections (R^2^ = 0.35, p < 0.0001), indicating that users became more efficient with continued use.
Wenceslao et al. (2019)^42^	Not specified - within 2019	Philippines	Usability evaluation study (using SUS)	Primary care	One GP	Not explicitly stated, but usability testing for the AIVT system itself	• Usability score by SUS • Transcription accuracy and latency	The AIVT system received a low usability score (45/100), indicating major usability challenges. Feedback included suggestions to incorporate keyword triggers for various consultation-note sections to facilitate documentation and reduce time spent on data entry.

OP, Out-Patient; PA, Physician Assistant; GPs, General Practitioners; SUS, System Usability Scale.

The AI tools used in the studies varied from commercial platforms (e.g., DAX by Nuance Communication, Google Cloud, and Amazing Transcribe) to customised applications and prototypes interfaced with local EHR systems, as demonstrated in Wang et al.^40^ All tools adopted Automatic Speech Recognition (ASR) technologies powered by deep learning and NLP to transform clinician-patient conversations into structured clinical documentation. The table in Appendix 4 specifies the AIVT tools used in each study and summarises their technical features.

### Quality assessment of the included studies

Of the nine included studies, three were assessed as having high methodological quality,^7^^,^^35^^,^^41^ four were of moderate quality,^37^, ^38^, ^39^ and two were assessed to have low methodological quality.^40^^,^^42^ Confounding bias was a concern across all studies due to the relatively unrealistic consultation settings during the assessments, such as quiet, controlled environments with uninterrupted communication and high-quality microphones. Selection bias was of concern in five studies, mainly due to participant recruitment methods that limited generalisability, such as only including native speakers or excluding patients with specific medical conditions, such as psychiatry and paediatrics.^37^^,^^39^, ^40^, ^41^, ^42^

Haberle et al. had moderate methodological quality but showed a risk of performance bias due to an understandable lack of blinding on AIVT use, given the nature of the human–system interaction assessment in the study.^36^ As participants were understandably aware of using AIVT tools, many of whom were self-nominated, there might be an inherent tendency toward greater engagement and preconceived expectations regarding the benefits of AIVT tools.^36^ The quantitative component of Islam et al. is subject to confounding and selection biases, limiting generalisability, as their evaluation was restricted to only patients with diabetes, despite their stated aim of developing an AIVT tool for broad clinical use.^41^

The two studies with a high risk of bias had distinct methodological limitations: one assessed AIVT technology in a single consultation with limited robustness.^42^ The other study invited two medical students familiar with digital tools to simulate consultations, limiting the findings’ applicability.^40^ The table in Appendix 3 summarises the MMAT risk of bias assessment outcomes for all the included studies.

### The impact of using AIVT on quality domains

#### Effectiveness

All studies (n = 9) highlighted the effectiveness of AIVT tools in documentation, enabling healthcare professionals to focus more on patient interaction. The automation of documentation helped reduce cognitive burdens and mitigate burnout among healthcare professionals.^38^ AIVT tools were able to capture key points during history-taking and, in most cases, achieved documentation quality comparable to manual transcription.^41^ In Goss et al.’s study, 75.5% of healthcare professionals reported fewer than 10 errors per transcription with 19.6% considering half or more of these errors clinically significant.^35^ Three studies noted some limitations in accurately capturing Non-Lexical Conversational Sounds NLCS and clinically significant information.^35^^,^^37^^,^^39^ Performance metrics such as Word Error Rate (WER), concept extraction, and F1 scores revealed mixed results, suggesting room for improvement in AIVT accuracy and reliability.^37^

To support the safe and effective use of AIVT tools, seven of the nine included studies explicitly reported providing training to participants, while the remaining two (Kodish-Wachs et al. and Tran et al.) implied the training component as both were conducted in simulated settings.^37^^,^^39^ Training styles varied across studies, including in-person sessions, virtual, and self-paced modules.

#### Efficiency

Five studies reported improved efficiency with AIVT for documentation,^35^^,^^36^^,^^38^^,^^40^^,^^41^ though one study also highlighted a potential risk of increased time spent reviewing consultation notes and correcting errors, occasionally leading to increased after-hours work.^36^ Time savings for healthcare professionals was the most commonly reported advantage over traditional methods like typing or dictation.^35^^,^^38^^,^^41^ Wang et al. found that a digital scribe was 2.7 times faster for history-taking and 2.17 and 3.12 times faster for physical examination documentation compared to typing and dictation, respectively.^40^ Efficiency gains were positively correlated with the number of encounters, as providers became more accustomed to the system.^40^

#### Safety

There was no clear consensus among the studies regarding the safety of relying on AIVT tools for clinical documentation without subsequent review by healthcare professionals. Three studies reported no safety risks,^36^^,^^38^^,^^41^ while three others raised safety concerns.^35^^,^^37^^,^^39^ Islam et al. noted AIVT’s potential to improve the clarity of prescriptions and medication instructions, which can improve care safety.^41^ On the contrary, Kodish-Wachs et al. highlighted concerns about transcription inaccuracies, especially with medication names, which could pose significant safety risks.^37^ Metrics such as error rates and their clinical implications were frequently highlighted, as they may lead to some safety risks.^35^^,^^37^

#### Patient-centredness

All six studies that referred to the influence of AIVT use on patient-centredness reported positive findings,^7^^,^^35^, ^36^, ^37^^,^^40^^,^^41^ primarily by facilitating a more personalised approach during consultations.^7^^,^^41^ AIVT enabled healthcare providers to focus more on patient interaction, leading to improving the overall consultation experience for both patients and providers.^7^^,^^35^ For example, Owens et al. reported that 80.9% (76.4–85.4%) of patients felt their consultations were more personalised when AIVT was used, as providers could engage more with them during appointments.^7^ Wang et al. highlighted the use of patient-centred communication techniques, such as signposting and summarising, alongside AIVT to create structured documentation, enhancing the quality of patient-provider interactions.^40^ Two studies raised ethical concerns about respecting patient autonomy, emphasising the need to inform patients about AIVT use and obtain consent to record their voices when necessary.^7^^,^^36^

#### Equity, integration, and timeliness

Equity concerns can be noted related to the limited generalisability of findings due to participant homogeneity in most studies. Although all included studies described their AIVT tools, whether commercially available or newly developed, as intended for general medical use, without specifying target populations or conditions, several evaluated their tools using selective inclusion or exclusion criteria. This raises potential equity issues regarding the technical and practical use of these tools. For instance, Owens et al. primarily included highly educated Caucasian patients,^7^ while Islam et al. focused exclusively on patients with diabetes.^41^ Wang et al. excluded certain groups, such as paediatric patients and patients with psychiatric disorders.^40^ Furthermore, most studies involved only native English speakers,^35^^,^^37^^,^^39^^,^^40^ and factors like patient volume and healthcare professionals’ familiarity with technology were identified as potential confounders.^35^^,^^36^^,^^38^^,^^41^

Integration of AIVT documentation outputs with EHR systems was observed in most studies to ensure seamless incorporation into patient records.^35^^,^^42^ Four studies reported seamless integration,^7^^,^^36^^,^^41^^,^^42^ whether experimental or practical, while others did not explicitly mention this. Haberle et al. reported a significant improvement in documentation completion rates within 24 h with AIVT users, highlighting the system’s potential to streamline documentation.^36^

## Discussion

### Main findings

This systematic review assessed the impact of AIVT systems on automating clinical documentation and their influence on the quality of care in primary care and outpatient settings. Effectiveness was identified in all nine studies, while patient-centredness and safety appeared in six, efficiency in five, integration in four, equity in three, and timeliness in two.

AIVT showed notable advantages in effectiveness and efficiency, improving documentation speed and reducing cognitive and administrative burdens.^35^^,^^36^^,^^38^^,^^40^ It also supported more patient-centred consultations by allowing providers to focus on active communication during appointments.^7^^,^^40^^,^^41^ Most studies reported feasible integration of AIVT with EHR systems, enhancing care coordination.

The quality of AIVT-generated documentation was often comparable to, and sometimes better than, traditional methods.^36^ However, unreviewed transcription inaccuracies, particularly with medication names, raised potential safety concerns,^37^ which could be exacerbated in real-world practice outside the controlled and monitored study conditions. Generalisability was also limited by selective patient inclusion, such as studies focusing only on patients with diabetes or excluding paediatric and psychiatric consultations.^39^, ^40^, ^41^

### Comparison with previous literature

Our findings align with Falcetta et al., ’s 2022 systematic review,^21^ which highlighted AIVT’s potential to improve consultation effectiveness and efficiency by reducing documentation burdens.^21^^,^^36^^,^^38^ Additionally, Falcetta et al. also noted limitations, including the need for large-scale studies and challenges in EHR integration.^20^^,^^21^ While our review, which included five studies from 2023 to 2024,^7^^,^^36^^,^^38^^,^^39^^,^^41^ found no reported EHR integration issues, further research is needed. Large-scale, long-term evaluations remain critical research priorities.^1^^,^^21^

Potential patient safety concerns, as consistently highlighted in the literature, arise when transcription inaccuracies go unreviewed by clinicians. Factors such as noisy environments, diverse accents, and complex cases can contribute to these inaccuracies and the subsequent patient safety risks.^2^^,^^43^ The controlled nature of most studies may limit their applicability to real-world practice.^13^^,^^37^^,^^41^ Previous research supports reviewing AIVT-generated documentation before finalisation to ensure accuracy, safety, and accountability.^13^^,^^43^ Additionally, publication bias should be considered, as studies reporting safety risks may be less likely to be published, leading to an incomplete understanding of potential challenges.^44^

A key benefit of automating documentation is its potential to create more patient-focused consultations.^7^^,^^45^ Increasing reliance on forms, templates, and digital tools often detracts from the core purpose of medical appointments, active patient-provider interaction.^46^ AIVT can help restore this focus by prioritising meaningful communication.^47^ While reviewing AIVT-generated notes may initially increase post-consultation workload,^38^ this challenge can be mitigated with training, familiarity, and workflow adaptations, ultimately balancing efficiency with patient-centred care.^18^^,^^40^^,^^41^

The impact of AIVT on care quality in primary care and similar settings remains an area in need of further research.^2^^,^^43^ Advocates emphasise its ability to automate documentation, framing it as one less task for healthcare professionals, thereby reducing clinician workload and enhancing patient care by allowing clinicians more time to focus on patient interaction.^48^^,^^49^ Critics, however, argue that it may limit opportunities for critical thinking, structured reasoning, and accountability in documentation.^43^ As AIVT and other digital tools evolve, their effects on quality domains must be carefully assessed and monitored to ensure they enhance, rather than compromise, care quality.^49^^,^^50^

### Strengths and limitations

This review’s methodology adhered to PRISMA guidelines for rigorous study selection, data extraction, and bias assessment.^25^ Clear inclusion and exclusion criteria focused on emerging AIVT technologies, ensuring substantial inter-rater agreement during screening. Findings highlight AIVT’s impact on care quality and safety, extending beyond documentation accuracy to the doctor-patient relationship, clinician burnout, workforce satisfaction, and integrated care, supporting resilient health systems.

However, limitations were noted. Study heterogeneity, including variations in AIVT tools, patient populations, and healthcare professionals, hindered comparability. For example, calculating a standardised Word Error Rate (WER) wasn’t feasible due to differences in AIVT software used, some being generative models and others trained on medical terminology, as well as inconsistencies in reported metrics. The limited scale of studies also posed concerns. For instance, Wenceslao et al. assessed their AIVT tool in a single consultation with one physician (35), and many studies relied on simulated consultations, limiting real-world applicability. The predominant focus on English-speaking, often native, populations further raises concerns about generalisability. Additionally, the fact that seven out of nine studies were conducted in the US highlights the need to assess AIVT use in diverse contexts.

It is also important to highlight that some results and reported outcomes from the included studies may overlap across multiple quality domains, reflecting the inherently interconnected nature of these domains in practice. For example, improving patient-centred communication through the use of AIVT tools during consultations may concurrently influence perceptions of safety and demonstrate effectiveness in achieving the intended functionality and use of these tools.^7^

### Implications for research, policy, and clinical practice

Considering the observed variation in implementation and the practical challenges to adopting AIVT tools in practice that were reported in the included studies, the findings suggest a need for developing standardised policies and regulations to guide AIVT integration into clinical practice at both system and facility levels to ensure contextual adaptation. Policymakers should establish evidence-based frameworks to ensure safety and reliability before widespread adoption.^2^ Policies must address ethical concerns, including data security, confidentiality, patient consent, and accountability for errors.^51^

Research should prioritise stringent safety standards and real-world testing across diverse patient populations and healthcare professionals with varying technological expertise. Large-scale, multi-centre trials in primary care and outpatient settings, including remote consultations, are needed to assess scalability and generalisability.^15^ While many studies focus on AIVT’s technical development, further research is required on its impact on patient outcomes, provider concerns, and workflows.^47^ Transparent reporting of methodologies and results, regardless of outcomes, is essential to mitigate publication bias and ensure a realistic evaluation of AIVT use.^44^

In clinical practice, healthcare professionals must receive adequate training before AIVT adoption and implementation, which was applied in all the included studies.^52^ Transparency about risks and mitigation strategies is crucial for patient safety.^18^^,^^52^ Workflow adaptations, including time for note review or troubleshooting, should be clearly defined and agreed upon to ensure smooth integration at each facility.^18^ It is also essential to understand clinicians’ expectations of this technology, including the expected level of automation, time required for text review, reliance on AIVT for further tasks such as drafting referral letters, and any additional technical requirements for its use in remote consultations.

It is essential that future research supports policymakers by providing evidence to inform the development of tailored protocols that ensure the safe, effective, and equitable adoption of AIVT within healthcare systems. While international frameworks, such as the WHO’s “*Guidance on Ethics and Governance of Artificial Intelligence for Health”*,^53^ offer broad strategic direction, the formulation of context-specific guidelines at the national or local level remains essential. A recent example is the guidance issued by the National Health Service (NHS) in England on the implementation of AIVT in health and care settings,^54^ which reflects the importance of aligning global principles with local needs and regulatory contexts.

### Conclusion

This review highlights AIVT’s significant potential to improve clinical documentation and patient experience of clinical consultations through enhanced efficiency, reduced clinician workload, and improved clinician-patient focus during consultation. AIVT-generated documentation in the included studies was generally comparable to or exceeded traditional methods, though transcription inaccuracies, especially with medication names, pose safety risks. However, controlled study environments and limited patient diversity restrict generalisability, highlighting the need for large-scale, real-world trials focusing on long-term safety, equity, and clinical outcomes. There is an urgent need for policymakers to establish protocols and guidelines to regulate the increasingly widespread use of AIVT tools among clinicians.

## Contributors

All authors contributed to the conceptualisation of the study. AA, ALN, and AD developed and reviewed the systematic review protocol. AA and RA conducted the initial data screening and quality assessment, and ALN provided senior advice when needed. AA and RA independently conducted data extraction and analysis, which were reviewed and discussed with ALN. AA, RA, and ALN had accessed and verified the underlying data, including the full-text manuscripts of the included studies. All authors contributed to and confirmed the interpretation of the results. AA drafted the initial full manuscript. ALN, HA, BH, RA, and AD reviewed and provided feedback on the manuscript. All authors read and approved the final version of the manuscript. The corresponding author confirms that all listed authors meet authorship criteria and that no others meeting the criteria have been omitted.

## Data sharing statement

All the relevant data are included in the manuscript and supplementary files.

## Declaration of interests

ALN and BH have been supported by the NIHR Applied Research Collaboration North-West London. BH also works as a Clinical Safety Officer for eConsult Health, which is a provider of an online consultation platform for primary, secondary, urgent and emergency NHS care. HA is also the Chief Medical Officer at Harbinger Health, which focuses on exploring new approaches to cancer screening, diagnosis and management. All authors declare no competing interests.
