# Supplementary material for: The impact of using AI-powered voice-to-text technology for clinical documentation on quality of care in primary care and outpatient settings: a systematic review
Source: eBioMedicine. 2025 Jul 21;118:105861. doi: 10.1016/j.ebiom.2025.105861 (PMC12301838; doi:10.1016/j.ebiom.2025.105861)
Supplement: Appendix 1 [file mmc1.docx]

# **Appendices**

**Appendix 1:** Search terms in databases

| **Medline**  (via OVID) | **AND** | speech recognition software/ or (("Automatic Speech Recognition" or (Voice technolog*) or "Conversational AI" or "Conversational Artificial Intelligence") AND (transcript* or document* or dictat* or report* or “note-taking”)) or digital scrib* or medical scrib* or clinical scrib* or (“speech to text” or “voice to text” or (Transcription Technolog*) or (Speech adj Transcrip*) or "Ambient Voice Technology") or ((‘‘voice’’ OR ‘‘speech’’) AND (recogni*) AND autom* AND docum*) |
| --- | --- | --- |
|  |  | exp artificial intelligence/ or “Artificial Intelligence” or "Machine Learning" or "Deep Learning" or "Natural Language Processing" |
| **Embase**  (via OVID) | **AND** | (exp automatic speech recognition/ or (("Automatic Speech Recognition" or (Voice technolog*) or "Conversational AI" or "Conversational Artificial Intelligence") AND (transcript* or document* or dictat* or report* or “note-taking”)) or (“speech to text” or “voice to text” or (Transcription Technolog*) or (Speech adj Transcrip*) or "Ambient Voice Technology") or digital scrib* or medical scrib* or clinical scrib* or ((‘‘voice’’ OR ‘‘speech’’) AND recogni* AND autom* AND docum*)) |
|  |  | (exp artificial intelligence/ or *machine learning/ or Natural Language Processing/ or “Artificial Intelligence” or "Machine Learning" or "Deep Learning" or (predictive adj analytic*) or "Natural Language Processing") |
| **Global Health** (via OVID) | **AND** | voice recognition/ or (("Automatic Speech Recognition" or (Voice technolog*) or "Conversational AI" or "Conversational Artificial Intelligence") AND (transcript* or document* or dictat* or report* or “note-taking”)) or ("Speech-to-Text" or “speech to text” or "Voice-to-Text" or “voice to text” or (Transcription Technolog*) or (Speech adj Transcrip*) or "Ambient Voice Technology") or ((‘‘voice’’ OR ‘‘speech’’) AND recogni* AND autom* AND docum*) |
|  |  | (exp artificial intelligence/ or machine learning/ or “Artificial Intelligence” or "Machine Learning" or "Deep Learning" or (predictive adj analytic*) or "Natural Language Processing") |
| **Scopus** | **AND** | "Speech to Text" or "Voice to Text" or “ambient voice technolog*” or “medical scrib*” or “clinical scrib*” or “digital scrib*” |
|  |  | {Artificial Intelligence} or {Machine learning} or {deep learning} or {Natural Language Processing} |
| **CINAHL** | **AND** | (MH "Voice Recognition Systems") or (("Automatic Speech Recognition" or "Voice technolog*" or "Conversational AI" or "Conversational Artificial Intelligence") AND (transcript* or document* or dictat* or "note-taking" or transcrib*)) or ("speech to text" or "voice to text" or (Transcription n1 technolog*) or "Ambient Voice Technology" or (medical n1 scrib*) or (clinical n1 scrib*) or (digital n1 scrib*)) |
|  |  | (MH "Artificial Intelligence") OR (MH "Artificial Intelligence, Generative") or (MH "Machine Learning") OR (MH "Deep Learning") or (Artificial Intelligen*) or "Machine Learning" or "Deep Learning" or "Natural Language Processing" |
