# Supplementary material for: The impact of using AI-powered voice-to-text technology for clinical documentation on quality of care in primary care and outpatient settings: a systematic review
Source: eBioMedicine. 2025 Jul 21;118:105861. doi: 10.1016/j.ebiom.2025.105861 (PMC12301838; doi:10.1016/j.ebiom.2025.105861)
Supplement: Appendix 2 [file mmc2.docx]

**Appendix 2:** Data extraction items

| **Item** | **Description** |
| --- | --- |
| Author | First author et al. |
| Title | Title of the published paper |
| Country | Where the study has been conducted |
| Publication date | As stated on the publishing journal |
| Date of study conduction | If it was not explicitly mentioned in the manuscript, an estimated period was identified based on the methodology. |
| Quantitative/Qualitative | Based on the methodology used and the data analysis section for each study |
| Study type | Considering the main study type if explicitly mentioned by the original study authors or as identified based on the methodology description. |
| Objectives | As stated in the published manuscripts |
| Settings | Whether primary care, hospital wards, outpatient clinics, or a mix of different settings. |
| Sample size | The overall number of participants and their specific characteristics if mentioned |
| Healthcare professional type | The speciality of the involved healthcare professionals in each study, including general practitioners or other specialities. |
| Patient categories | Specifying if the study involved or excluded specific patient groups, such as diabetic patients, paediatrics, or other. |
| Intervention | The type of the intervention used in the study, in terms of which AIVT tool was used. |
| Consultation type | Specifying if primary care, outpatient, emergency, or virtual versus in-person consultation. This item also specifies if it was simulated, live, or recorded consultations. |
| Control/comparison group | Specifying if the intervention used in the study has been compared to any other intervention or a control group. |
| Methods | Summarising the study methodology |
| Outcome measures | Enumerating the outcome measures for each study |
| Confounding factors | Listing the clearly stated or identified confounding factors. |
| Key findings | Listing the key findings of each study, whether quantitative or qualitative. |
| Quality domains | Reflections on each of the seven quality domains of interest, including the six domains of the IOM and the seventh (i.e., integration) added based on WHO publications. |
| Source of funding | If explicitly mentioned. |
| Conflicts of interest | If explicitly mentioned |
| Ethical considerations | Identifying potential ethical considerations for each study and the use of the intervention AIVT tool for each study. |
| Notes | General notes for each study, including the need for staff training on using different AIVT tools. |
