# Supplementary material for: The impact of using AI-powered voice-to-text technology for clinical documentation on quality of care in primary care and outpatient settings: a systematic review
Source: eBioMedicine. 2025 Jul 21;118:105861. doi: 10.1016/j.ebiom.2025.105861 (PMC12301838; doi:10.1016/j.ebiom.2025.105861)
Supplement: Appendix 3 [file mmc3.docx]

**Appendix 3:** Quality assessment using the MMAT tool

| **Quantitative**  **non-randomised** | **Participant Representativeness** | **Measurement Appropriateness** | **Outcome completeness** | **Confounder Adjustment** | **Intervention Consistency** | **Overall Quality** |
| --- | --- | --- | --- | --- | --- | --- |
| Owens et al. | Yes | Yes | Yes | No | Yes | High |
| Tran et al. | No | Yes | Yes | No | Yes | Moderate |
| Owens et al. (2) | Yes | Yes | No | No | Yes | Moderate |
| Wang et al. | No | No | Yes | No | Yes | Low |
| Kodish-Wachs et al. | No | Yes | Yes | No | Yes | Moderate |
| Islam et al. | No | Yes | Yes | No | Yes | Moderate |
|  |  |  |  |  |  |  |
| **Quantitative observational** | **Sampling Relevance** | **Sample Representativeness** | **Measurement Appropriateness** | **Nonresponse Bias** | **Statistical Appropriateness** | **Overall Risk Evaluation** |
| Wenceslao et al. | No | No | Yes | N/A | No | Low |
| Goss et al. | Yes | Yes | Yes | No | Yes | High |
|  |  |  |  |  |  |  |
| **Mixed methods** | **Rationale for Design** | **Component Integration** | **Integration Interpretation** | **Addressing Inconsistencies** | **Methodological Quality** | **Overall Risk Evaluation** |
| Islam et al. | Yes | Yes | Yes | Yes | Yes | High |
|  |  |  |  |  |  |  |
| **Qualitative** | **Approach Appropriateness** | **Data Collection Adequacy** | **Data-Derived Findings** | **Interpretation Support** | **Coherence of Methods** | **Overall Risk Evaluation** |
| Islam et al. | Yes | Yes | Yes | Yes | Yes | High |
|  |  |  |  |  |  |  |
| **Randomised controlled** | **Randomisation** | **Group Comparability** | **Outcome Completeness** | **Blinding of Assessors** | **Intervention Adherence** | **Overall Risk Evaluation** |
| Haberle et al. | Yes | No | Yes | No | Yes | Moderate |
