# Supplementary material for: The impact of using AI-powered voice-to-text technology for clinical documentation on quality of care in primary care and outpatient settings: a systematic review
Source: eBioMedicine. 2025 Jul 21;118:105861. doi: 10.1016/j.ebiom.2025.105861 (PMC12301838; doi:10.1016/j.ebiom.2025.105861)
Supplement: Appendix 4 [file mmc4.docx]

Appendix 4: A summary of the AIVT tool(s) used in each study

| **Study** | **AIVT tool name** | **Brief description of the AIVT technology** |
| --- | --- | --- |
| Goss et al. (2019) | Dragon® 10.1 and Dragon® Medical 360 | Front-end speech recognition medical dictation software integrated with Epic EHR. Users dictate directly into the EHR with real-time transcription. |
| Haberle et al. (2024) | DAX™ (Dragon Ambient eXperience) by Nuance Communication, Inc. | Ambient voice technology combining speech recognition, NLP, and AI to automatically document patient-provider conversations. |
| Islam et al. (2024) | AssemblyAI (Custom digital scribe and prescribe system) | Web-based integrated system using LSTM model and AssemblyAI for speech-to-text conversion, with automated prescription generation capability. The system includes audio recording, text processing, medical term extraction, and automated documentation in SOAP format. |
| Kodish-Wachs et al. (2018) | 1. Bing Speech API (V1)  2. Google Cloud Speech API (V1)  3. IBM Speech To Text (V1)  4. Azure Media Indexer (V1)  5. Azure Media Indexer 2 Preview (V2)  6. Nuance.SpeechAnywhere (V3.2)  7. Amazon Transcribe Preview (V1)  8. Mozilla DeepSpeech (V0.1) | Comparative study of eight commercial and open-source speech recognition engines applied to clinical conversations. All applied cloud streaming, except Mozilla DeepSpeech that was streaming locally. All of these speech recognition systems include Automatic Speech Recognition (ASR) technology powered by deep learning models. They typically share core components like acoustic modeling, language modeling, and speech-to-text transcription systems, often trained on large datasets to support real-time or batch transcription from different sources. |
| Owens et al. (2) (2024) | DAX™ (Dragon Ambient eXperience) by Nuance Communication, Inc. | Ambient voice technology combining speech recognition, NLP, and AI to automatically document patient-provider conversations. |
| Owens et al. (2024) | DAX™ (Dragon Ambient eXperience) by Nuance Communication, Inc. | Ambient voice technology combining speech recognition, NLP, and AI to automatically document patient-provider conversations. |
| Tran et al. (2023) | Google Cloud Speech-to-Text ("medical_conversations" model) and Amazon Transcribe Medical ("primarycare" + "conversation" model). | Commercial cloud-based speech recognition services with specialised medical language models designed for multi-speaker clinical conversations. |
| Wang et al. (2021) | Custom digital scribe prototype | Academic prototype utilising Google Speech-to-Text for transcription combined with custom NLP to generate documentation based on patient-centred communication patterns. |
| Wenceslao et al. (2019) | Custom EMR plugin using:  1.IBM Watson Speech to Text.  2. HTML5 Web Speech API.  3. cTAKES | Custom EHR plugin for MyLifeEMR combining speech recognition services with clinical text processing (cTAKES) to generate structured SOAP notes. |

**Abbreviations in the table:**

- LSTM: Long Short-Term Memory
- “V”: software version
- NLP: Natural Language Processing
- EHR: Electronic Health Records
- API: Application Programming Interface
- SOAP: Subjective, Objective, Assessment, and Plan
